# MENTAL HEALTH AND CAREGIVING SELF-EFFICACY AS PREDICTORS OF PERCEIVED OVERALL HEALTH AMONG ADRD CARE PARTNERS

**DOI:** 10.1093/geroni/igad104.3466

**Published:** 2023-12-21

**Authors:** Scott Wilks, Laura Ainsworth, Catherine Lemieux, Youn Kyoung (Lily) Kim, Alisha Thompson, Rhonda Bardales, Julia Trahan

**Affiliations:** Louisiana State University, Baton Rouge, Louisiana, United States; Louisiana State University, Baton Rouge, Louisiana, United States; Louisiana State University, Baton Rouge, Louisiana, United States; Louisiana State University, Baton Rouge, Louisiana, United States; Louisiana State University, Baton Rouge, Louisiana, United States; Louisiana State University, Baton Rouge, Louisiana, United States; Louisiana State University, Baton Rouge, Louisiana, United States

## Abstract

A geriatrics workforce enhancement program piloted interdisciplinary telehealth services to Alzheimer’s disease and related dementias (ADRD) care partners. The team in medicine, nursing, and social work noted that consistently mentioned mental health issues and social isolation. The purpose of this study was to evaluate the extent to which the care partner’s sense of caregiving self-efficacy, juxtaposed with the aforesaid mental health issues, predict the care partner’s sense of their overall health. In a hospital setting in southern United States, the team collected data from 95 ADRD care partners on demographic characteristics and on measures of loneliness, anxiety, depression, caregiving self-efficacy, and perceived overall health. Data were analyzed using OLS multiple regression. Sample characteristics served as covariates. Adjusted R-squared estimated the corrected goodness-of-fit. The sample’s average age was approximately 62 years. Most identified as female (74%) and Caucasian/white (66%); about one-third identified as African American/Black. The plurality reported as adult child of the care recipient (47%). An overwhelming majority (94%) scored as clinically anxious; 29% scored as clinically depressed; 33% reported loneliness; 12% reported low caregiving self-efficacy; and 20% reported poor overall health. Depression and loneliness negatively predicted caregivers’ overall health (B=-.17, p<.001; B=-.15, p<.05, respectively). Surprisingly, anxiety positively predicted caregivers’ overall health (B=.54, p<.001). Self-efficacy was not a significant predictor. Adjusted R-squared value was .63. Results underscore the importance of including mental health and social networks when evaluating and treating ADRD care partners’ overall health. Future studies should consider broader, diverse samples to determine the reliability of these findings.

